# Targeted sequencing in the loblolly pine (*Pinus taeda*) megagenome by exome capture

**DOI:** 10.1186/1753-6561-5-S7-O48

**Published:** 2011-09-13

**Authors:** Leandro Neves, John Davis, Brad Barbazuk, Matias Kirst

**Affiliations:** 1 Plant Molecular and Cellular Biology Program, University of Florida, Gainesville, FL, USA; 2 Plant Molecular and Cellular Biology Program and School of Forest Resources and Conservation, University of Florida, Gainesville, FL, USA; 3 Plant Molecular and Cellular Biology Program and Department of Biology, University of Florida, Gainesville, FL, USA

## Background

An essential use of genomics is in the discovery of genes controlling complex, quantitative traits. In forestry, attempts to identify genes that regulate quantitative variation are still limited to a few Association Studies (AS) focused largely on candidate genes [1]. In most studies, few markers have been identified in association with quantitative traits.

Recent advances in DNA sequencing create the potential for high-throughput SNP genotyping at low cost, by re-sequencing genomes of interest [2]. For the particular case of conifers, two obstacles remain: (i) the lack of a reference genome to align the DNA sequences and identify SNPs, and (ii) the size and complexity of the genome that hinders the *de novo* assembly of reads. Whereas whole-genome sequencing of a large number of conifer genotypes is still unfeasible, concentrating the sequencing on gene-rich regions is an alternative to generate markers that are more likely to capture variation associated to complex traits. Here we report our approaches to develop methods of genotyping based on whole-exome capture using in-solution target enrichment (Agilent’s SureSelect) followed by high-throughput sequencing (Illumina’s GAIIx).

## Methods

### Probes for target enrichment

To capture a defined genic portion of the *Pinus taeda* genome we designed 54,773, unique, 120-mer RNA probes that tilled across a collection of 14,729 EST assemblies (unigenes). For 3,615 genes we could predict gene models and avoid probes on known exon-intron junctions.

### Genetic material, library preparation and sequencing

A total of 72 seeds collected from a female tree (10-5 genitor) were used for library preparation. Haploid megagametophyte tissue was dissected and DNA was extracted using standard protocols. Libraries were prepared as recommended by Illumina, with some custom modification of concentration, adapter sequence and fragment size selection. Custom barcoded adapters were used to pool up to eight genotypes in a single hybridization, which followed Agilent’s SureSelect protocol. Captured-libraries were sequenced using GAIIx with 115nt single-end run.

## Results and discussion

### Preliminary validation

To test the potential of target enrichment in pine we prepared sequencing libraries from three different genetic complexities from single genotypes/haplotypes: haploid cDNA from megagametophyte (n=12), haploid DNA from megagametophyte (n=12) and diploid DNA extracted from needles (2n=24). After hybridization of these libraries to SureSelect probes, we cloned each captured library into vectors and sequenced 96 fragments using Sanger sequencing. After reads were aligned to the reference sequences used for probe design (BLAST e-value=1x10^-10^), 70, 84 and 80% of the quality-filtered reads from haploid cDNA, haploid DNA and diploid DNA aligned uniquely to a unigene, respectively. Therefore, sequence capture is highly specific, even when the complexity of the material increases.

### Pipeline development for mapping population

We modified the sequence capture protocol to increase throughput by constructing libraries from haploid DNA of 72 megagametophytes in 96-well plates. An average of 25.9 million single-end reads were obtained from flow-cell channel containing 8 barcoded megagametophytes, for a total of 56 megagametophytes sampled across 7 channels lanes. The remaining two pools (16 individuals) have not been sequenced. Despite multiplexing a large number of megagametophytes, we detected low variation in read numbers between haplotypes within a multiplex reaction (Figure 1). Due to the lack of a reference genome, bioinformatics experiments were designed to test whether the best approach to identify polymorphisms would result from aligning the sequences directly to the unigene sequence using Mosaik (http://bioinformatics.bc.edu/marthlab/Mosaik), or by alignment of reads to a new reference sequence defined by *de novo* assembly of the captured reads with the ABySS [3]short read sequence assembler.

The analysis using Mosaik compared different levels of polymorphism between reads and the unigene sequences resulting from alignments over a range of mismatch tolerances (from 1% to 50%). Figure 2A shows that the number of reads that aligned to a single region of the reference (i.e. uniquely aligned) increases linearly until 10% of mismatches in the alignment are accepted, and stabilizes after that. Interestingly, the percentage of reads non-uniquely aligned remains lower than 2%, regardless of the mismatch rate. Next we tested if accepting higher mismatch rates adds more reads to the same genes, or if genes previously not captured are now represented. Again, at a 10% mismatch rate (Figure 2B) the total number of genes with at least one read aligned reaches its maximum, with 22% of the reads aligning to more than 10,000 genes.

Analyzing the data using a *de novo* assembly approach does not appear to be more suitable. ABySS assemblies were performed utilizing a range of k-mer values (50 to 95, increments=5) and the contigs formed were analyzed. As expected, the number of contigs drops considerably as a function of the k-mer size (Figure 2C). To evaluate the quality of contigs generated at each k-mer, we compared them to the unigene sequences (BLAST identity≥85%) and counted the number of genes represented by at least one contig. This number decreased as a function of the k-mer size (Figure 2D); and, within the same genotype the sequence assemblies generated using a high k-mer were already presented in the assembly using a lower k-mer. However, when comparing two genotypes assembled with the same k-mer value about 30% of the genes are unique to each genotype. This suggests that additional sequencing is necessary for an adequate analysis of the efficiency of sequence capture.

We are currently sequencing the same libraries from both ends (i.e. paired-end sequencing) to increase coverage and depth. This sequencing data is expect to identify additional SNP markers for segregation analysis, and to help define the sequencing requirements to confidentially perform target-enrichment resequencing and genotyping in complex genomes.
